# From accepting to distancing as different coping strategies in persons with young onset Parkinson’s disease

**DOI:** 10.1038/s41531-026-01336-5

**Published:** 2026-04-04

**Authors:** DHB Speelberg, TF Peerbolte, WM Kapelle, C van der Heijden, BR Bloem, B Post, MJ Meinders

**Affiliations:** 1 Department of Neurology, Radboud university medical center, Donders Institute for Brain, Cognition and Behaviour, Center of Expertise for Parkinson and Movement Disorders, Nijmegen, The Netherlands; 2 Department of Medical Psychology, Radboud university medical center, Nijmegen, The Netherlands

**Keywords:** Diseases, Health care, Psychology, Psychology

## Abstract

People with young onset Parkinson’s disease (YOPD) encounter unique challenges. Coping is therefore vitally important to effectively manage stressors. We thematically analyzed 17 semi-structured interviews regarding coping strategies. In cohort data (YOPD *n* = 74, late onset PD *n* = 214), we analyzed the frequency of coping styles. Most people with YOPD used a mixture of five styles: (1) taking action, (2) distancing, (3) mental solutions, (4) social support, and (5) coming to terms. Within these styles, we identified 28 different strategies, and described how participants apply these in a flexible manner. We found an association between a higher use of coping through distancing and psychological distress and used qualitative interview data to explore a possible bidirectional relationship. This study offers a first indication of how people cope with YOPD. The observed association between distancing and distress should be studied further. If found to be causal, coping flexibility might become a potential interventional target.

## Introduction

Parkinson’s disease (PD) is a neurodegenerative disorder with an alarmingly increasing prevalence^1^ across both younger and older persons^2^. Most individuals are aged 65 or older when diagnosed with PD^3^. Estimates of proportions for those receiving their diagnosis between the age of 21 and 50, defined as Young Onset Parkinson’s Disease (YOPD)^4^, vary from 3 to 10%. The disease in persons with YOPD manifests differently in terms of clinical presentation, motor complications and imaging characteristics compared to those diagnosed beyond the age of 50^5,6^. Moreover, psychological distress is common: depressive and anxious symptoms are often reported in individuals with YOPD^7–9^. Comparisons between people with YOPD and people with a later disease onset provide conflicting results, with a trend for more depression in YOPD, but no difference for anxiety^10–14^. YOPD also interferes at a distinct stage of life with consequences on multiple domains^15^; for instance, one might consider having children, maintaining a young family or advancing a professional career at the time of diagnosis. As a result, coping with a diagnosis of YOPD and its consequences can be enormously challenging, yet adequate coping is vitally important as a basis for moving forward after the diagnosis.

Coping is defined as the process of cognitive and behavioral efforts to manage stressors. These stressors might be internal or external and are appraised to be taxing or exceeding an individual’s resources^16^. Coping can explain why a similar stressor, event or disease has a completely different impact on the way individuals experience these. Certain coping styles, such as distraction and emotion-focused coping, are associated with a higher burden of disease^17^. Coping is a dynamic process related to its appraised stressor and the context. The individual’s ability to adapt their coping styles, termed coping flexibility, can be critical for effective coping^18^.

In general, people with PD cope with the consequences of disease by striving for normality, focusing on the present, staying independent and avoiding challenges^19^. However, insights about coping in YOPD with its unique features and stage-of-life related challenges remain limited. As an individual’s distress can be influenced by coping strategies^20^ and because coping strategies are flexible, insights into an individual’s coping strategy can be a starting point for tailored therapy aimed at reducing distress^21,22^. Therefore, a deeper understanding of coping is relevant to better support individuals with YOPD.

This study aimed to gain insights into coping styles and coping flexibility in individuals with YOPD. Furthermore, we investigated coping styles and distress in both persons with YOPD and Late Onset PD (LOPD), operationally defined as being diagnosed after the age of 70^12^.

## Results

### Participant characteristics

17 participants were included for the interview study and 288 participants for the cohort study (Table 1). Participants were highly educated compared to the general population. This was especially pronounced in the interview study with 47% having completed education on university level.

**Table 1:** Participant characteristics

	Interview study	Cohort study	
Characteristics	Participants *n* = 17	YOPD *n* = 74	LOPD *n* = 214
Gender, n (%) women	7 (41.2)	42 (56.8)	69 (32.2)
Age at diagnosis, years, mean (SD)	41.3 (5.3)	45.4 (4.9)	74.9 (4.2)
Disease duration, years, mean (SD)	6.9 (4.2)	12.0 (8.4)	3.5 (2.5)
Highest level of education completed n (%) Vocational level Higher professional level University level Unknown	4 (23.5) 4 (23.5) 8 (47.1) 1 (5.9)	41 (55.4) 22 (29.7) 11 (14.9) 0 (0.0)	101 (47.2) 69 (32.2) 41 (19.2) 3 (1.4)
Employed, n (%) yes	7 (41.2)	24 (32.4)	8 (3.7)
In a relationship, n (%) yes	15 (88.2)	57 (77.0)	177 (82.7)

Overview of demographical characteristics. SD=standard deviation, n=number of participants.

### Cohort study

Results on coping and distress in persons with YOPD and LOPD are presented in Table 2.

**Table 2:** Coping and psychological distress outcomes in YOPD and LOPD, cohort study

Outcome	YOPD (*n* = 74)	LOPD (*n* = 214)	p-value
**WCQ; Coping style**, relative score, % (SD)			
Taking action Distancing Goal oriented Seeking social support Avoidance and acceptance	28.02 (15.92) 27.85 (17.40) 47.97 (22.49) 42.16 (18.83) 32.81 (16.66)	26.36 (15.41) 27.07 (14.96) 43.30 (21.32) 35.95 (20.50) 37.15 (19.06)	0.4282 0.7144 0.1103 0.0226 0.0825
**Most prominent coping style**, *n* (%)			
Taking action Distancing Goal oriented Seeking social support Avoidance and acceptance	2 (2.7) 4 (5.4) 39 (52.7) 19 (25.7) 10 (13.5)	9 (4.2) 17 (7.9) 88 (41.1) 42 (19.6) 58 (27.1)	0.7346 0.6079 0.0837 0.2722 0.0177
**Depression (BDI)**	**YOPD (*n*** = **74)**	**LOPD (*n*** = **213)**	
- Sum score (mean, SD) - Minimal severity, *n* (%) - Mild severity, *n* (%) - Moderate severity, *n* (%) - Severe severity, *n* (%)	12.88 (7.55) 42 (56.8) 20 (27.0) 9 (12.2) 3 (4.1)	11.86 (6.34) 138 (64.8) 49 (23.0) 22 (10.3) 4 (1.9)	0.2606 0.2183 0.4855 0.6616 0.3792
**Anxiety (STAI)**	**YOPD (*n*** = **74)**	**LOPD (*n*** = **213)**	
- State, mean (SD) - Trait, mean (SD)	37.8 (11.00) 38.6 (10.00)	38.8 (10.54) 38.7 (9.50)	0.4735 0.9446
**Acceptance of Illness (AIS)**	**YOPD (*n*** = **74)**	**LOPD (*n*** = **214)**	
- Sum score, mean (SD)	26.05 (6.18)	26.39 (5.53)	0.6669

*BDI* Beck’s Depression Inventory, *STAI* State Trait Anxiety Index, *AIS* Acceptance of Illness Scale, *WCQ* Ways of Coping Questionnaire, *YOPD* Young Onset Parkinson’s Disease, *LOPD* Late Onset Parkinson’s Disease.

Persons with YOPD and LOPD used “goal oriented and planful problem solving” most often as a coping style (Table 2). Persons with YOPD deployed “seeking social support” significantly more often than persons with LOPD (*p* = 0.0226). Among the YOPD participants 90.5% deployed all five main styles, while 95.9% used at least four styles. In participants with LOPD, 89.7% used all five styles, and 97.2% used at least four. People with YOPD and LOPD did not differ on any of the psychological distress scales.

In addition, we investigated the association between coping style usage and measures of psychological distress (supplementary materials, Table S1). In the univariable analysis, higher usage of “distancing and fantasizing” was consistently associated with higher psychological distress for both persons with YOPD and LOPD: it was associated with more severe depressive and anxiety symptoms, and a lower degree of acceptance. In the multivariable analysis, these associations remained significant. Moreover, for individuals with YOPD, a higher usage of “seeking social support” was associated with less severe anxiety symptoms and a higher degree of acceptance, and “taking action and emphasizing the positive” was associated with a higher degree of acceptance in the multivariable analysis. For individuals with LOPD, “goal oriented and planful problem solving” was associated with less severe depressive and anxiety symptoms, and higher degree of acceptance, however this association was less pronounced. Coping styles explained between 11.67 and 37.44% of the variance in psychological distress. Additional analyses in model 2 and 3 (supplementary materials, Table S2), including group (YOPD or LOPD) and disease duration, showed no significant changes in associations between coping and distress.

### Interview study

**Fig. 1:**
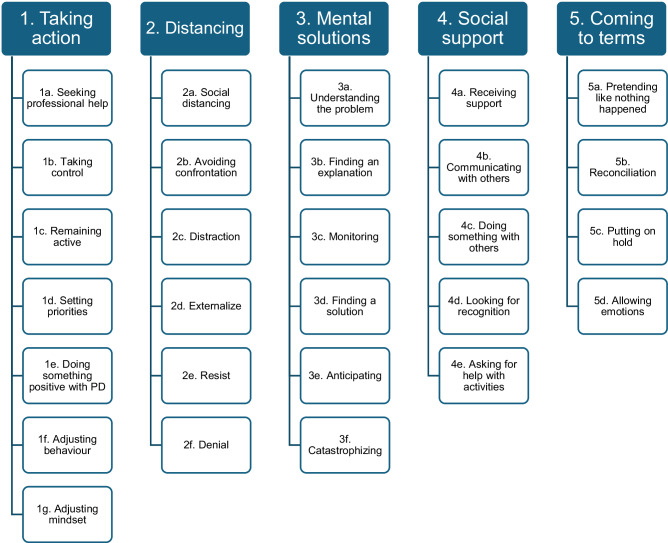
*Overview of themes of coping in YOPD.* Coping styles are displayed as themes with a blue background, coping strategies are displayed below as subthemes with a white background.

Through thematic analysis, we initially formulated 31 coping strategies in a total of 9 themes. During group discussion with the supervisory team combined with iteratively checking the interviews, we merged four themes that did not originally fit in the five styles of the framework with their respective main style, reasoning from the core motivation of each coping strategy. This resulted in a total of 28 coping strategies within the five main styles of the framework. Participants of the focus group concluded these findings to be relatable and comprehensive. An overview of the styles and strategies is provided in Fig. 1. Style names were shortened for brevity and to better reflect their description based on the content. Transcript quotations to substantiate these findings are presented in Table 3. Below, we elaborate on two coping strategies for each coping style; to illustrate how coping strategies are being applied in daily life of a person with YOPD. A full description of all strategies is included in the supplementary materials.

**Table 3:** Transcript quotations

Theme	Illustrative quote
**I. Taking action**	Active coping style; efforts to improve the situation
Seeking professional help	“Accepting Parkinson is definitely something. I’ve struggled with it, I would lie if I said otherwise. I also went to see a medical psychologist. They helped me a lot.” (M, 52Y)
Taking control	“He [the treating traumatologist] thought of post-traumatic stress disorder, I thought of Parkinson. […] But I insisted on further investigation due to my profession. And I did get that diagnosis.” (M, 49Y).
Remaining active	“Because I still really enjoy working. It triggers you. You need it to do. If you don’t do anything, you’ll lose it. And if you – “ *What would you lose in that case?* “The – Being busy with what you’re doing. […] Just, being busy. And if you don’t do that, you’ll go sit on a chair, and things really won’t improve from that. “ (M, 47Y)
Doing something positive with PD	“It makes you feel a bit useful towards the illness. What I notice — in myself, but I think others feel the same — it does help if you have the disease, if you can help it along a bit. That is also the reason why I’m happy to do this. I like helping other people by sharing my experiences.” (M, 44Y)
Setting priorities	“And I think that due to the Parkinson, but also the meningitis, so the whole process of being ill, it has led me to be less stressed or rushed, I guess, and that I’ve learned to relativize certain things and think more about where my actual priorities are?” (F, 51Y)
Adjusting behavior	“You’ve got sufficient ingredients to first start tinkering yourself to see whether you can influence the illness with it. So I started being more fanatic in yoga. I changed my diet. I started exercising a lot more. I started taking rests a lot more. I started scaling down my secondary duties.” (M, 52Y)
Adjusting mindset	“Well, you know, I had already read a lot about it by then. Yes, it may sound a bit silly, but I’m actually somewhat happy that it has got a name. I don’t see it as a sentence, but more like an instruction manual? Like, okay, I really need to approach my life differently now. […] And looking back, some things started to make sense, too.” (M, 47Y)
**II. Distancing**	Active and passive coping style; prevent having to deal with PD
Social distancing	“…So I went home early because I was moving all over the place. That was really upsetting. People were really watching. They thought, oh my god. And I struggled with that, but my friends do as well.” (F, 48Y)
Avoiding confrontation	[about fears for the future] “No, I just don’t think about it. It would be a pity to let it influence my quality of life. […] If I think about everything that might happen, I might as well stop living now.” (M, 35Y)
Distraction	“…everybody’s life keeps going and you feel like your life just… stops at that moment. And yes, that sucks. […] when things don’t go the way I hoped, I start creating things. […] I start making flower arrangements, or…” (F, 51Y)
Externalization	“I did that the first year for a long time. I’d get up in the morning, and thought, if I could make a wish, I would wish it [the disease] away. And it has taken me a long time to say, this does not go away.” (F, 55Y)
Resistance	“The disease is the only thing on your mind, trying to solve it, or maybe even prove that it is not really there.” (M, 49Y)
Denial	“So we’re talking about, what a five year gap, when you […] sort of deny that you are ill.” *You also did not feel like you were a patient?* “No, definitely not. And yes, some things did get more difficult, you know.” (M, 41Y)
**III. Mental solution**	Active coping style; proactive, planning ahead, coming up with mental workarounds to decrease the stress from PD
Understanding the problem	“Some even say: once you google ‘Young Onset Parkinson Disease’, your life is over. […] No, be sure to find people that really know their stuff, and understand your situation. And go and ask them questions or discuss problems. Don’t do it on google, it won’t make you happy.” (M, 49Y)
Finding an explanation	“Every now and then, when things got a bit tense or even very calm, I had trouble with buttons with my right hand, or a slight tremble when writing. So I thought, that’ll be because of the accident [in medical history]. […] And my arm swing decreased, […] I also dislocated my shoulder flying a plane or skiing years before.” (M, 49Y)
Anticipating	“If you know that your bladder doesn’t always work on time, you make sure that you carry the things you need, so you can deal with it when something goes wrong. And you make sure that you arrive early, to make sure that you’ve got time to arrange things if something goes wrong.” (F, 51Y)
Monitoring	“And when I can’t answer a math problem I start thinking: hey, why can’t I solve this? Is this – is it a genuinely hard problem, or am I just not getting it right now?” (M, 35Y)
Finding a solution	“For example, this morning, my daughter – she needed a dictionary. And she completely panicked. And that affects my body. So I think, let’s calm down. I tell her we’ll fix it. So I contacted somebody, do you have that dictionary? So I rush over there on my bike…” (F, 44Y)
Catastrophizing	“I used a walker, or whenever I visited the city with my friends, maybe two or three times a year, I went with them in a wheelchair. But when I went with my husband to the garden center, you have these wheelchairs, I sat in those chairs.” *Was it primarily anxiety, or did your legs hurt, or were you tired?* “Fear. And suddenly, you realize, you are way more dependent.” (F, 48Y, only 2 years after initial diagnosis)
**IV. Social support**	Active and passive coping style; relying on others to decrease stress experienced from PD together
Receiving support	“I have a very solid support network surrounding me, I can really trust and build on them. They always said [participant’s name], something’s off, but we don’t know what. My parents always said this, too. But in the end, we’ll stand with you, or stand behind you, however you say.” *Yes, so you felt very supported?* “Yeah definitely, otherwise I’d basically have no life left, right.” (F, 48Y)
Communicating with others	“I think the main message I’d like to share, is to not let it get you down. Be open about the disease. Because that will bring you a whole lot. At least, it did for me. And I feel like it prevents you from getting isolated, too.” (M, 44Y)
Doing something with others	“I always did something active on my birthday, like making paper kites, or a multipart competition.” *Simply doing something that’s dynamic?* “Yes […], and that’s something I’d like to pick up again, but I also have my own thoughts about it – I would not want to join just any choir, but at one point, the music school offered this small choir. And I felt like yeah, that would be manageable for me, stimulus-wise.” (F, 48Y)
Looking for recognition	“What I enjoyed most of being in that setting, was that you could talk about it with peers. How do you see this or that? How are you dealing with that on the job?” *Did you learn things, or what did you get from it?* “Yeah, in general. Yeah, learning?” *Or recognizing things?* “Yes, recognition, and discussing how he or she copes?” (M, 45Y)
Asking for help	“I mean, you have to drive for 2 h to get here. According to the planning, we are talking for two hours, and afterwards I need to drive 2 h back home.” *That is really a lot*. “And it’s too much. So I’ll ask her [participant’s daughter]. And she helps me. She goes – she will join me on the way.” (M, 58Y)
**V. Coming to terms**	Passive coping style; “path of least resistance”, trying to invest little effort to deal with the stressful situation
Pretending like nothing happened	“I tried to carry on as usual. […] But yeah, some things just don’t work anymore.” (INT47)
Reconciliation	“What are you even resisting? There’s nothing to resist, it’s in your head and it really doesn’t come out. It’s more the situation itself, you know? Like I said, the anger, the sadness. You start to think how, and why – and there’s also a lot still unknown about how you got the disease, and how it will develop. But I told myself, as long as even the experts don’t have an answer, how should I know?” (F, 55Y)
Putting on hold	“And at that moment, you get the diagnosis […] so much information. All things I could not do anymore, information everywhere. […] And I said – I said, I need to process this a little. Can’t we talk about all that information in the next appointment? […] She [neurologist] said, well, that is a good idea.” (F, 48Y)
Allowing emotion	“For me, everything is a process. Acceptance is, too. And I know myself, I’m in that process now, and part of it is being angry. […] And part of it is being sad, too.” (M, 49Y)
**Coping Flexibility**	Process about coping, reflecting about it and adjusting if applicable.
Abandonment	“I have been really strict about it [lifestyle], like drinking no more alcohol, things like that. But now, I’ll have a beer every now and then. Not as much as I used to – I’m getting older. But you know, it’s about being able to do things you enjoy.” (M, 49Y)
Re-coping	“Because I was afraid that I could not do those things anymore. I used to tennis a lot, but I stopped. We always went skiing, but I did not go anymore. Now, however, I’m doing all those things again, because, you know, I’m not as good as I used to be, but I still have just as much fun. So you know, that’s a change I’ve gone through.” (F, 55Y)
Meta-coping	“If I keep on going [working] like this without any adjustments, I honestly expect I’ll hit a burn-out. […] Because I know – I mean, I burned out 15 years ago for very different reasons, and I learned a whole lot from that.” (M, 44Y)

For each quotation, participant’s gender and age at the time of the interview are shown. Interviewer comments are presented in italic.

### Taking action

Taking action is an active coping strategy, with efforts to positively change the situation. Often, these efforts are directed at the problem at hand, and therefore, it is a predominantly problem-focused coping style.

Participants coped by actively taking control of their situation. Some participants did this by asking for a second opinion in cases of doubt, or taking initiative in discussing the timing for starting or increasing their medication regimen. Others stated their resistance to starting with medication due to their wish to stay independent, a fear of harmful effects, or uncertainty about the positive effect of the medication, causing them to actively steer away from this. Moreover, participants took control in their working environment by setting clear boundaries with their employer and pro-actively suggesting work-related solutions. Lastly, individuals took control in their social environment through addressing situations that are undesirable for them and discussing how their environment can deal with these situations to better suit the participants.

Second, adjusting one’s mindset was a prominent strategy of taking action. One method was “positive reframing”; putting a positive spin on something that initially was perceived as negative, such as thinking of the diagnosis as a manual to better understand their limitations and deal with daily challenges. Focusing on the positive was also common; some participants noted getting closer with family, being able to enjoy “the little things in life”, or generally living a more conscious life compared to before. For some, this also included reflecting on one’s identity to be more than just a successful professional career. Moreover, the use of humor was noted by participants as a coping strategy, making it easier to speak about their hardships. Lastly, participants showed endeavors to exert cognitive control; they addressed their inner selves when they focused too much on negative thoughts or reassured themselves that a period of pain will eventually end. One participant noticed that negative thoughts worsened the (non)motor complaints, triggering him to actively shift the attention to the positive side of the situation.

### Distancing

Distancing as a coping style includes mostly active ways of reducing the degree of having to deal with PD and can be both emotion- or problem-focused.

People with YOPD used social distancing as a coping strategy, by staying away from social events to avoid confrontation, shame or stimuli. Dyskinesias were a reason for social distancing because individuals felt ashamed and believed their environment was ashamed of them. In addition, participants consciously avoided talking about PD to prevent others feeling sorry or misunderstanding them. Other reasons for social distancing included, e.g., that they do not want to be a burden to others, and avoiding shocking others with the diagnosis and hardships they endure. Lastly, social distancing included hiding symptoms, for example holding onto their arm to hide tremors.

Another distancing strategy was to avoid confrontation with their disease. Specifically for persons with YOPD, going to a peer event with a general, often older population with PD, frequently led to feelings of alienation and subsequent avoidance of confrontation with advanced disease. Additional rationales included being anxious of hearing symptoms they had not yet experienced and may - or may not - experience in the future. Some participants actively avoided activities that emphasized their limitations, causing them to, e.g., buy peeled vegetables, or not driving themselves but relying on family instead. Besides that, participants frequently put up cognitive barriers, simply not wanting to think about PD every single day, avoid thinking about the future and its uncertainty, or to not give in to emotions such as feelings of sadness.

### Mental solutions

Mental solutions are a (pro)active coping style about understanding the problem, coming up with solutions and anticipating on problems in the future. These strategies typically presented themselves in this sequential order.

Participants put effort into understanding the problems they encountered. An important aspect of this was to look for information on the internet, in books, but also by talking to peers. Specifically, some participants expressed their preference for information by peers, as this was more catered towards young persons and often more reliable than a search on the internet. They wanted this information to better understand the disease, medication, and prognosis. Looking for information remained important in all stages of the disease. Sometimes information seeking led to distress instead of providing reassurance. For instance, one participant in the diagnostic phase found that brain tumors are an indication for brain imaging, which was insufficiently explained by their physician, causing anxiety. Information was especially sought for major decisions such as starting with medication or starting with advanced therapies.

When thinking about and preparing for the future, some participants lost themselves in a negative train of thoughts, termed catastrophizing. One such spiral of negativity is demonstrated by a participant developing fear of falling in the early phase of disease. This caused her to avoid walking altogether, resulting in using a wheelchair in the first years of disease. After discussing this with a peer, she overcame her fear and started training to walk long distances, like pilgrimages. This confirmed that there was in fact no somatic reason for her to be limited to a wheelchair. Other participants were convinced that their disease would prove fatal soon, based on stories of much older patients. Some even started arranging their own funeral, believing their death to be imminent.

### Social support

The social support included both passive and active ways of coping and was often emotion focused. Many participants stating feeling supported by their social circle, of which partners played a key role. However, friends, family, peers, colleagues, healthcare providers and teachers at a child’s school are all mentioned as sources of social support.

On the one hand, receiving support was a passive coping strategy. Participants received support through people being there for them, helping them to reflect on their disease limitations and offer perspective, or sharing sadness. Some participants felt motivated by others to overcome their fears or to get active during moments of apathy or (social) anxiety.

On the other hand, participants applied this strategy more actively through initiating conversation with others about their situation. Topics included both physical and non-motor symptoms, and the timing varied for when participants felt comfortable to talk about this. Specifically, some found support in reaching out to peers and finding recognition. Furthermore, they actively asked for support with activities. This included asking for practical help in household tasks, in transport when mobility was decreased, or asking their families for help managing a day out with their children.

### Coming to terms

Coming to terms is a passive style of coping, aimed at regulating themselves instead of solving the problem at hand, and therefore an emotion-focused coping style. It is often the path of least resistance to deal with a stressful situation.

One such strategy is ignoring the situation or pretending like nothing happened. To continue living their life as usual, participants stated that they ignored selective symptoms or the diagnosis altogether.

Another strategy is reconciliation. Participants rarely used the word “acceptance” due to the progressive nature of PD; with the continuous threat of decline of function or manifesting of new symptoms, there was no perceived constant state that could be accepted. Some participants explained that they had accepted some aspects of disease, such as the fact that it is incurable, the uncertainty of the future, having to quit work, or diminishing of relationships. Furthermore, participants stated not being bothered anymore by bystanders staring, or not feeling shame anymore when they experienced dyskinesias. Participants recognized their lack of control in such situations.

### Coping flexibility

The majority of participants deployed different coping strategies over the years, suggesting they demonstrated coping flexibility. Others described their coping as relatively rigid. Coping flexibility involves abandoning one coping style and often replacing it with another (re-coping). This process requires reflection on the effectiveness of coping (meta-coping)^23^.

Abandonment is one of the components of coping flexibility. For instance, a participant initially coped by trying to understand the problem, but stopped gathering information due to the negative consequences of being confronted with a much more advanced stage of disease. Other examples include letting go of a strictly healthy lifestyle because this decreased their joy in life, or to stop wishing that the disease would go away.

Often subsequently to abandonment, participants demonstrated re-coping. One participant illustratively described her attempts to regain a sense of control over the unpredictability of her disease by initially adhering to a consistent and structured daily schedule. When this did not work, she shifted attention to her medication regimen and the meals she had with them. At the same time, she discussed the problem with peers and received additional tips. Realizing the limited benefit of all this, she stopped trying to regain control and accepted the unpredictability of her disease instead. Another illustrative example was a participant starting to do a lot of activities again for fun, while she initially avoided these activities out of fear.

Throughout the interviews, many participants showed meta-coping by reflecting on their experiences with coping. This was beneficial for some, whilst others experienced insecurity or regret because of this. One part of meta-coping is contemplation about coping and its effectiveness in the past, and making conscious decisions based on this for the future. To aid in this contemplation, some used past experiences such as a burnout, whilst others reached awareness through family conversations, or with the help of a psychologist. Furthermore, this increased awareness resulted in a shift in the way they handled stressors. Reflecting on coping in the past resulted in assessing certain coping strategy as undesirable, which in turn led participants to abandon these coping strategies in similar situations in the future. Some noted that a predominantly rational way of dealing with PD was not helpful, whilst others stated that they thought they should reframe the way they saw their diagnosis, identity or need for medication into something less negative. Likewise, some participants evaluated coping strategies as being desirable in certain situations, affirming their belief and intention to cope this way. Participants realized for instance that sharing thoughts and emotions with others had a positive impact instead of keeping these to themselves and continued to do so.

Additional analyses on differences between coping strategies between participants, based on characteristics like gender, duration of disease and education level, revealed no evident pattern. This suggests that coping strategies were applied evenly and flexibly, regardless of the characteristics.

### Integration analysis

To better understand the quantitative association between a higher usage of “distancing” and more psychological distress, we explored the relationships between moments of distancing and psychological distress in the interviews.

One participant recognized feelings of loneliness, apathy and sadness from a previous depressive episode in life. This time, it coincided with avoiding any confrontation with PD. This suggests co-existence of both distancing and distress at that time. Furthermore, participants demonstrated how distress led to distancing. They described that feelings of sadness, isolation, anxiety, and an inability to accept the disease caused them to avoid confrontation with the disease. In contrast, one participant showed how distancing can lead to distress: she elaborated on her experience, stating feelings of anger, sadness and hopelessness whilst resisting the disease, which diminished after choosing to be less avoidant. Therefore, adjusting the coping style away from “distancing” was a conscious decision resulting in less distress. On the contrary, some participants made a deliberate decision to avoid or reduce thinking about the disease, moving towards a “distancing” style, because this contributed to a greater sense of well-being and being able to enjoy the present.

## Discussion

We explored the coping strategies deployed by persons with YOPD. They were more likely to seek social support than those with LOPD. Furthermore, most individuals used a mixture of coping styles simultaneously, depending on the circumstance. Many participants displayed reflective capabilities to switch between different coping strategies, adjusting to the situation at hand. Despite numerous challenges introduced by the disease, positivity in coping was prevalent, but distancing and catastrophizing were also used. Lastly, we found an association between applying “distancing” as a coping style and higher levels of distress.

Positivity in the face of adversity was common, with positive reframing of a debilitating diagnosis and looking for positive ways to support others. This phenomenon is described as “silver linings”; a range of positive experiences including a better focus in life, or improved relationships^24^. Quantitative results support this observation, indicating that persons with PD score higher on optimism than on pessimism^25^. Optimism may be psychologically protective, being associated with lower levels of distress, either by mediating the role between coping and depressive symptoms^25^, or through more direct pathways^26^. These findings reminded us of the plea made for receiving a dose of “hopamine” through a personalized form of hope and optimism regarding the future^27^.

Distancing was a prominent coping style in our cohort, in line with a meta-synthesis^19^ that reported “avoiding unpredictable challenges” as one of three main coping strategies, with components of social distancing. We show in our exploratory analysis that use of distancing may be associated with elevated levels of psychological distress. While in line with earlier work suggesting that distancing may lead to more distress, our approach does not allow us to infer causality. Specifically, emotion-focused coping strategies are associated with greater psychological distress^17,25,28^. Furthermore, individuals endorsing an external locus of control, as part of a distancing coping style, tend to report higher levels of distress^29^. However, our interview study suggests that this relationship may be bidirectional (i.e., distress may also lead to more distancing) and is personal. These findings highlight the need for further work to examine the nature of the observed association, also at the individual level. A better understanding of the association between distancing and distress may potentially hold clinical value; for example, identification of frequent use of distancing could serve as a signal for further psychological assessment, particularly for symptoms of depression and anxiety.

Our results also highlight the need for clinical vigilance on catastrophizing. This coping strategy was described previously, with people with PD stating impending doom, hopelessness and being afraid to die prematurely after receiving the diagnosis^30^. In persons with PD experiencing pain, catastrophizing is associated with presence of non-motor symptoms and lower quality of life. It also mediates the relationship between younger age and lower scores on quality of life^31^. If these observed associations could be proven to be causal, then catastrophizing could become a targetable factor that negatively contributes to quality of life, and perhaps especially so among younger patients. However, this earlier work was performed in a general PD population and warrants further investigation in persons with YOPD. In our study, catastrophizing led individuals to avoid actions that, in turn, could have positively influenced their quality of life, such as physical activity. In these cases, catastrophizing might offer a target for improving care and quality of life.

Both people with YOPD and LOPD frequently utilized “use of social support” as a coping strategy. In a meta-synthesis on coping in PD, social support was the only theme consistently reported in all 14 included studies^19^. People with YOPD more often deployed social support as a coping strategy than those with LOPD, confirming previous findings^32^. Still, YOPD participants encounter difficulties in accessing appropriate forms of social support. For instance, many YOPD participants reported a lack of recognition in patient support groups, as they themselves are a minority within the broader PD population. They described a disconnection in terms of disease stage and stage of life. Participants actively sought recognition from peers in a similar stage of life, who also had to deal with comparable stressors such as young children, work and finances. These qualitative findings align with a quantitative survey study on unmet psychosocial needs in YOPD, which reported a pronounced demand for age-matched peer communities^33^.

In contrast with previous literature^34^, our results demonstrate within-person flexibility in coping strategies. Whereas previously coping was assumed to be stable, as a personality trait, interviewees in our study adjusted their coping strategies when deemed necessary. These contrasting findings might be explained by the research methods, as qualitative research provides more opportunities to capture subtle nuances or mechanisms. Likewise, stability in coping over time was demonstrated at the group level, but flexibility was shown at an individual level^35^. A similar dynamic aspect of coping was recently demonstrated using focus groups^36^. However, whilst those authors found a staged and sequential evolution in coping, we found that participants shifted more freely, based on the stressor at play and previous experiences.

Coping flexibility might offer an interesting possible target for future intervention. Higher scores on coping flexibility have been associated with lower scores on distress^35,37–39^, and coping flexibility can improve through interventions based on mindfulness^40^ or cognitive behavioral therapy^41,42^. We therefore feel that coping flexibility is a potential target of interest. Further research is warranted to examine the association between flexibility and mental health outcomes in this population.

Our study presents explorative results in a previously understudied population. The findings were based on a combination of qualitative and quantitative data. The framework on coping offered a theoretical background, and our purposive sample ensured representation of a broad spectrum of coping strategies. We further enhanced analytical rigor by discussing the results with a focus group of people with YOPD. Involvement of a patient researcher aided us to maintain results, theme descriptions and word choice grounded in real life.

Our study is not without limitations. First, given the explorative design, no definitive conclusions can be drawn on the role of coping styles and strategies. For instance, disease severity, heterogeneity and medication use were not included in the quantitative analyses, although this might influence experienced stressors, resources available and coping preferences. We did correct the analyses using disease duration as partial surrogate for disease severity; this did not affect the associations between coping and distress. Second, interviews were conducted at one time point, requiring participants to recall events from years before. This likely introduced some recall bias. However, a retrospective description of coping remains interesting, given that time provides participants the opportunity to process complex events and reactions, like finding meaning in a stressful moment. This ultimately determines the impact at the present^43^. Nonetheless, a future ethnographic study investigating momentary coping in key milestones of disease, such as receiving the diagnosis or starting an (advanced) treatment, would be worthwhile. Third, the use of a theoretical framework as a starting point for the analysis may have introduced confirmation bias. However, we limited this risk by continuous awareness for this risk during the analysis and repeated discussions of findings with our diverse research team. Moreover, we believe that the rich description of the coping styles is the main outcome of our analysis. Fourth, the findings on coping flexibility in our qualitative data are of exploratory nature. Future research on this topic using quantitative scales, such as the improved Coping Flexibility Scale-Revised^23^ could expand our insights on this topic. Finally, the high education level in our sample, compared to the general population, might have affected the results. Of note, a recent study found a higher socio-economic status among PD patients across the Netherlands^44^. Interestingly, in the interview study, participants with high educational levels showed no apparent differences in coping patterns compared to participants with a lower educational level. Nevertheless, qualitative data are not well suited for between-group comparison, so further research in disadvantaged populations is warranted.

This study provides new insights on coping in persons with YOPD. We demonstrate how this population applies flexibility in their coping. The association between distancing and distress warrants further research on this topic given the impact on the individual.

## Methods

### Study design

In a convergent mixed-methods design, we used triangulation of qualitative and quantitative results to enhance our understanding of patients’ experiences with coping. We investigated the associations between coping and distress quantitatively in a cohort of persons with YOPD and LOPD. Furthermore, we deepened our insights into the way coping is deployed by persons with YOPD through in-depth interviews. Methods are described in accordance with the COREQ-criteria^45^. We refer to the quantitative part as the cohort study, and to the qualitative part as the interview study.

### Participants

Participants in the cohort study took part in the PRIME-study^46^. Participants were recruited through ParkinsonNEXT, the Dutch Parkinson Patient Association, the annual ParkinsonNet congress and at the outpatient clinic. Inclusion criteria included a self-reported clinical diagnosis of parkinsonism, having visited a community hospital neurology outpatient clinic at least once in the year before inclusion and being able to provide written consent. Persons with drug-induced parkinsonism were excluded. We selected 74 participants with YOPD (diagnosed between ages 21–50), and 214 participants with LOPD (diagnosed at age 70 or older). We chose this age gap based on previous literature^12^ and to create a clear contrast between the two groups. Participants who did not complete the revised Ways of Coping Questionnaire (WCQ) were excluded from our analysis.

The interview study was part of a broader study on the impact of YOPD on daily life. We included persons being diagnosed with PD (confirmed by a neurologist) between the age of 21 and 50. Participants were recruited through convenience sampling given the low incidence and prevalence of YOPD, followed by snowball sampling. They were recruited at the Radboudumc outpatient clinic, through the Dutch Parkinson Patient Association and among participants of other clinical studies who had granted permission to be approached for research purposes. Inclusion criteria included being fluent in Dutch and being able to provide written consent. Recruitment was stratified for disease duration and took place between September 2020 and April 2021. Selection for this interview study was stratified to ensure disease duration variability (groups of 0–4 years since diagnosis, 5–9 and 10 or more) and representative gender distribution (men: women ratio of 3:2)^9^.

### Data collection

We used baseline data collected in the PRIME-study. For coping we used the revised WCQ^18^, surveying how often certain aspects of coping were deployed. We used the validated Dutch 67-item version^47^. Several aspects of psychological distress were investigated; depression was assessed through the Beck Depression Inventory-II (BDI-II)^48^, anxiety through both the state and trait component of the State-Trait Anxiety Index (form Y version, abbreviated STAI)^49^ and acceptance was investigated through the Acceptance of Illness Scale (AIS)^50^.

We collected participant characteristics (demographics, employment status and medication use) of the interview study through a structured telephone questionnaire. Single semi-structured interviews were conducted thereafter by an independent journalist (IM), trained in journalism. The interviews took place in a studio in the Radboudumc, ensuring that no other stakeholders were present and participants could speak freely. Audio was recorded and field notes were taken during the interview. Interview transcripts were not returned to the participants prior to analysis. Interview duration varied between 62 and 124 min. Themes in the interview guide were based on a mind-map created by a person with YOPD^15^. The interviews were structured chronologically, starting with the period surrounding the diagnosis and working up to the present, ending with the participant’s view on the future. Being an integral part of the impact of a chronic diagnosis, coping was a recurrent theme and discussed with each participant. The interview guide was refined iteratively as the interviews were conducted. The final version is included in the supplementary materials.

### Data analysis

Summary measures for demographics in the cohort study were calculated for both groups through means (standard deviations) and proportions. For the analysis, we used the five coping styles as derived from a previous factor analysis of the WCQ in the PRIME cohort, including “taking action and emphasizing the positive”, “distancing and fantasizing”, “goal oriented and planful problem solving”, “seeking social support”, and “avoidance and acceptance”^51^. Scores on items belonging to the same coping style were summed and normalized, resulting in comparable relative scores for each style ranging 0–100%. Furthermore, the most prominent coping style, i.e., the style with the highest relative score, was determined for each individual. Results were pooled and compared between participants with YOPD and LOPD.

For the BDI-II, STAI and AIS, mean scores for both YOPD and LOPD were calculated. Additionally, BDI-II scores ranging from 0 to 13 were classified as minimal depressive symptoms; 14–19 as mild; 20–28 as moderate and 29–63 as severe and proportions were calculated^48^. For all comparisons we used independent t-tests and chi-squared tests, depending on the variable characteristics, with a critical p-value of 0.05.

Furthermore, we performed univariable and multivariable regression analyses to investigate the relationship between coping and distress. Dependent variables in these analyses were BDI-II, STAI-State, STAI-Trait and AIS sum scores. Independent variables in the multivariable models were all five coping styles. We determined β-values and p-values for the independent variables and considered *p* < 0.05 as a significant association.

To explore associations in more detail, two independent variables were added in a step-wise forward manner: first, in model 2, the group-variable “YOPD or LOPD” was added and subsequently, in model 3, “disease duration” (in years). All statistical analysis was performed using the statistics program R, version 4.4.1.

In the interview study, audio tapes were transcribed verbatim. The resulting transcripts were analyzed using a reflexive thematic analysis due to its exploratory nature, its flexibility, and the ability to provide outcomes that can be transferable to the patient population^52^. Transcripts were first read repeatedly to familiarize ourselves with the data. Two of the authors (DHBS – male MD and PhD-student and TFP – female PhD-student) coded the first six transcripts independently and consensus was achieved through open discussions. Subsequent transcripts were coded by DHBS individually. Periodically additional transcripts were coded by both authors to monitor whether consensus was still met, and quality remained high. Data saturation was assessed after the first 15 transcripts, adhering to the concept of being a degree instead of a determinate timepoint^53^. At this point, data saturation was not yet met. After including two additional transcripts, no new themes or subthemes were defined, and the authors concluded that data saturation was met. Themes were referred to as “coping styles”, and subthemes were referred to as “coping strategies”. The previously defined WCQ factors were used as a starting framework^51^. However, we first coded the interview data in an inductive manner. During the analysis, we aligned the constructed themes with the WCQ factors, while being reflective and critical on the fit and allowing new themes to be constructed. In this approach we recognized that the predefined WCQ factors were based on a data-driven factor analysis instead of theory-based. Themes were discussed with the whole research team and refined accordingly. Subsequently, defined themes were checked for internal homogeneity and external heterogeneity.

In addition to the coping style framework, we applied the concept of coping flexibility. This consists of three components: (1) abandonment, a conscious decision to stop using a certain style of coping; (2) re-coping, devising a new way of coping with an identified stressor and (3) meta-coping, which is reflection on past coping efforts^23^. For the member-check we organized a focus group to discuss the results with a random selection of the interview sample included in the analysis (*n* = 6). Coding and analyses were performed using ATLAS.ti, version 24.

According to a convergent mixed methods design, quantitative and qualitative results were analyzed in parallel. Following this, consistent patterns in the cohort study prompted further investigation in the interview study. This combination of findings resulted in narrative integration of data^54^.

### Reflexivity

The author’s team consisted of a mix of male and female researchers and MDs. DHBS is a male MD and PhD-candidate. Having no prior experience with qualitative research, he followed two qualitative research courses. Weekly meetings were held during the coding and analysis phase with a team of experienced senior researchers (MJM and BP). Memos were used to document the analysis progress. A patient researcher (male, 54 years, diagnosed at the age of 48) was involved in the refinement phase of the themes and in the writing of this manuscript. There was no prior relationship between the participants, interviewer, and coding authors.

### Ethical considerations

This study was conducted adhering to the ethical guidelines of the Declaration of Helsinki (version 2013). All participants provided written consent. Ethics approval by the local ethical board at the Radboudumc was obtained for the interview study (file number 2022-15817). Furthermore, ethical approval for the quantitative part was obtained through the same ethical board (file number 2019-5618).

## Supplementary information

Supplementary materials

## Data Availability

Due to the sensitivity of the interview data and the restrictions in the informed consent, the data will not be stored at a public repository. The cohort data was made available by the PRIME research group. They were used under restrictions and are therefore not publicly available. Requests for access to the PRIME cohort can be submitted to the last author and will be reviewed by the PRIME research group.
