# Supplementary material for: From accepting to distancing as different coping strategies in persons with young onset Parkinson’s disease
Source: NPJ Parkinsons Dis. 2026 Apr 4;12:138. doi: 10.1038/s41531-026-01336-5 (PMC13249954; doi:10.1038/s41531-026-01336-5)
Supplement: Supplementary file 1 — Supplementary materials [file 41531_2026_1336_MOESM1_ESM.pdf]

From accepting to distancing as different coping strategies in persons with young onset Parkinson disease – Supplementary materials

Contents:

Table S1: Univariable and multivariable regression analyses, cohort study
Table S2: Multivariate regression analysis, cohort study, model 2 and 3

Supplementary note 1: Final version interview guide in English, interview study
Supplementary note 2: Full description subthemes, interview study

Table S1. Univariable and multivariable regression analyses, cohort study

	YOPD (n=74)					LOPD (n=213)				
	**Uni**	**p-value**	**Multi**	**p-value**	**Adjusted R^2**	**Uni**	**p-value**	**Multi**	**p-value**	**Adjusted R^2**
**Association with BDI**										
Taking action	0.001	0.988	-0.063	0.253		-0.022	0.445	-0.038	0.208	
Distancing	0.232	< 0.001	0.275	< 0.001		0.147	< 0.001	0.178	< 0.001	
Goal oriented	0.036	0.365	0.010	0.775		-0.026	0.208	-0.048	0.033	
Seeking social support	0.002	0.974	-0.084	0.061		-0.012	0.576	-0.020	0.356	
Avoidance and acceptance	0.014	0.789	0.031	0.529	0.2951	0.043	0.059	0.035	0.108	0.1616
**Association with STAI-S**										
Taking action	0.020	0.809	-0.078	0.346		-0.026	0.576	-0.022	0.656	
Distancing	0.306	< 0.001	0.370	< 0.001		0.253	< 0.001	0.319	< 0.001	
Goal oriented	0.047	0.414	0.007	0.893		-0.068	0.047	-0.127	0.001	
Seeking social support	-0.010	0.883	-0.129	0.056		0.007	0.841	0.003	0.930	
Avoidance and acceptance	0.070	0.369	0.093	0.207	0.2487	-0.017	0.649	-0.035	0.315	0.1819
**Association with STAI-T**										
Taking action	0.032	0.666	-0.040	0.570		-0.008	0.844	-0.017	0.689	
Distancing	0.325	< 0.001	0.389	< 0.001		0.254	< 0.001	0.309	< 0.001	
Goal oriented	0.037	0.485	-0.008	0.869		-0.041	0.186	-0.097	0.004	
Seeking social support	-0.006	0.918	-0.131	0.023		0.014	0.659	-0.001	0.977	
Avoidance and acceptance	0.025	0.725	0.037	0.550	0.3388	-0.016	0.644	-0.037	0.235	0.199
**Association with AIS**										
Taking action	0.037	0.417	0.102	0.019		-0.008	0.754	-0.034	0.198	
Distancing	-0.190	< 0.001	-0.242	< 0.001		-0.081	0.001	-0.112	< 0.001	
Goal oriented	-0.019	0.555	-0.006	0.837		0.052	0.004	0.082	< 0.001	
Seeking social support	0.019	0.629	0.084	0.016		0.020	0.279	0.016	0.418	
Avoidance and acceptance	-0.028	0.529	-0.061	0.106	0.3744	-0.015	0.442	-0.009	0.650	0.1167

Legend: Associations between coping styles and sum scores for depression, anxiety and acceptance. Both univariable(Uni) and multivariable (Multi) regression analysis were conducted, and β-coefficients are presented for each variable. Statistical significance is demonstrated through p-values, adhering to significance if $p < 0.05$. Adjusted R^2 values are presented for each multivariable model. A negative value suggests that higher usage of the coping style is associated with lower degree of depression, anxiety, or a higher degree of acceptance. Abbreviations: YOPD = Young Onset Parkinson Disease; LOPD = Late Onset Parkinson Disease; BDI = Beck Depression Inventory; STAI = State Trait Anxiety Index; STAI-S = STAI, State subscale; STAI-T = STAI, Trait subscale; AIS = Acceptance of Illness Scale.

Table S2: Multivariate regression analysis, cohort study, model 2 and 3; age of onset as group variable and disease duration

	Model 2			Model 3		
	**Multi**	**p-value**	**Adjusted R^2**	**Multi**	**p-value**	**Adjusted R^2**
**Association with BDI**						
Taking action	-0.047	0.070		-0.045	0.089	
Distancing	0.207	< 0.001		0.206	< 0.001	
Goal oriented	-0.030	0.120		-0.027	0.155	
Seeking social support	-0.036	0.069		-0.034	0.079	
Avoidance and acceptance	0.031	0.114		0.028	0.161	
LOPD	-1.425	0.083	0.2028	-0.346	0.742	
Disease duration	NA	NA		0.122	0.105	0.2074
**Association with STAI-S**						
Taking action	-0.036	0.399		-0.032	0.443	
Distancing	0.325	< 0.001		0.324	< 0.001	
Goal oriented	-0.082	0.008		-0.079	0.011	
Seeking social support	-0.031	0.329		-0.029	0.352	
Avoidance and acceptance	-0.012	0.716		-0.015	0.630	
LOPD	0.716	0.588	0.186	1.914	0.261	
Disease duration	NA	NA		0.136	0.264	0.1864
**Association with STAI-T**						
Taking action	-0.024	0.521		-0.019	0.602	
Distancing	0.326	< 0.001		0.325	< 0.001	
Goal oriented	-0.067	0.014		-0.063	0.021	
Seeking social support	-0.033	0.240		-0.031	0.269	
Avoidance and acceptance	-0.025	0.378		-0.030	0.284	
LOPD	-0.093	0.936	0.2286	1.587	0.287	
Disease duration	NA	NA		0.190	0.075	0.2334
**Association with AIS**						
Taking action	0.003	0.894		-0.001	0.977	
Distancing	-0.148	< 0.001		-0.147	< 0.001	
Goal oriented	0.053	0.002		0.050	0.003	
Seeking social support	0.033	0.056		0.031	0.068	
Avoidance and acceptance	-0.013	0.443		-0.009	0.611	
LOPD	0.668	0.356	0.1462	-0.750	0.417	
Disease duration	NA	NA		-0.161	0.016	0.161

Legend: Associations between coping styles and sum scores for depression, anxiety and acceptance. Multivariable analysis were conducted in a step-wise fashion: Model 2 included the group-variable "YOPD or LOPD". Model 3 additionally included "disease duration" (in years). β-coefficients are presented for each variable. Statistical significance is demonstrated through p-values, adhering to significance if $p < 0.05$. Adjusted R^2 values are presented. A negative value suggests that higher usage of the coping style is associated with lower degree of depression, anxiety, or a higher degree of acceptance. Abbreviations: YOPD = Young Onset Parkinson Disease; LOPD = Late Onset Parkinson Disease; BDI = Beck Depression Inventory; STAI = State Trait Anxiety Index; STAI-S = STAI, State subscale; STAI-T = STAI, Trait subscale; AIS = Acceptance of Illness Scale.

Supplementary note 1: Final version interview guide, interview study (translated from Dutch)

Interview guide Young Parkinson

1. Diagnosis

- What were your first symptoms?
- Do you remember receiving the diagnosis? What were your thoughts? Was it out of nowhere?
- What happened next? What were your thoughts? What worried you the most?
- Did you have a partner at the time of diagnosis? How did he or she respond?
- How did your family experience it?
- How did your friends respond?
- To what extent did you experience support from your social environment?
- How did the diagnosis impact your self-image?
- Did you immediately feel like a patient? Why? Did the way your environment reacted to it, play a role in this?
- How did you deal with the diagnosis? Did you use a certain strategy for that?
- Did you receive any psychological support? If so, how did you experience that? If not, did you have a need for it?

2. Life in the present with Parkinson's

- How is your experience living with Parkinson's in the present?
- What area does the disease affect the most? What's on your mind? (cognitive dysfunction, losing a partner, being taken seriously)
    - How does Parkinson's affect your family life? Your relationship with your partner? How does that relationship change, and how do you feel about that? How does your partner feel about that? (Are role patterns changing?)
    - How do your children experience it? How does that relationship change, and how do you feel about it? How do they feel about that?
    - What is the impact on making social connections? On your friends? On social isolation?
    - What is the impact on your sense of freedom? And your zest for life?
    - What is the impact on your self-image, or identity?
    - Does your social environment affect the way you experience the disease? Do you encounter misunderstanding, stigma or taboos?
    - To what extent does the disease affect your joy in life?
    - To what extent does the disease affect your sense of time?

- How does the disease affect your work?
    - Do you have any adjustments at work? Was it difficult to discuss?
    - Does your work affect your self-image? Does it provide anchor, or structure?
    - Do interactions with colleagues change?
    - How do you bring structure to your day? Is structure important to you?
    - Did losing the structure of daily work have a big impact on you?
    - To what extent is your work important for your identity?

- Finances
    - Do you worry about financial matters, like mortgage? How significant are these worries, compared to other domains of life?

- Psychological wellness
    - Do you notice any psychological changes? What are the signs? How does that affect you and your close social circle?
    - To what extent does the disease affect your mood?
    - Does the disease affect your confidence?
    - Did you have sufficient support in this? What did you miss?

- Physical limitations
    - To what extent do physical complaints contribute to your overall burden? What is most cumbersome? In what way does that interfere with your life at the moment?

- Healthcare and support
    - At this moment, what do you need the most?
    - How do you experience support and guidance in the hospital, and in other places? Does your partner or family get involved?
    - What is missing in support and guidance? What could be improved upon?
    - Which question do you feel hasn't been asked enough?
    - Did you get in touch with other persons with Parkinson's? Why, or why not? If so, what does this contact provide you?
    - How are you treated at this moment? Why?

- Coping
    - How do you cope with the disease? Have you accepted it? With what word would you describe this yourself?

3. Future

- When thinking about the future, what causes the most concern?
- How do you make plans of the future, or do you refrain from doing this? Do you only think about the short term future?
- Are you afraid of cognitive decline? Do you monitor yourself on it all the time?
- Do you hope to be cured?
- How do you provide meaning to your life? Do you experience a lack of purpose?
- Does the disease diminish your sense of time, or the experience thereof?

Supplementary note 2: Full description subthemes, interview study

Figure S1. Overview of themes of coping in YOPD

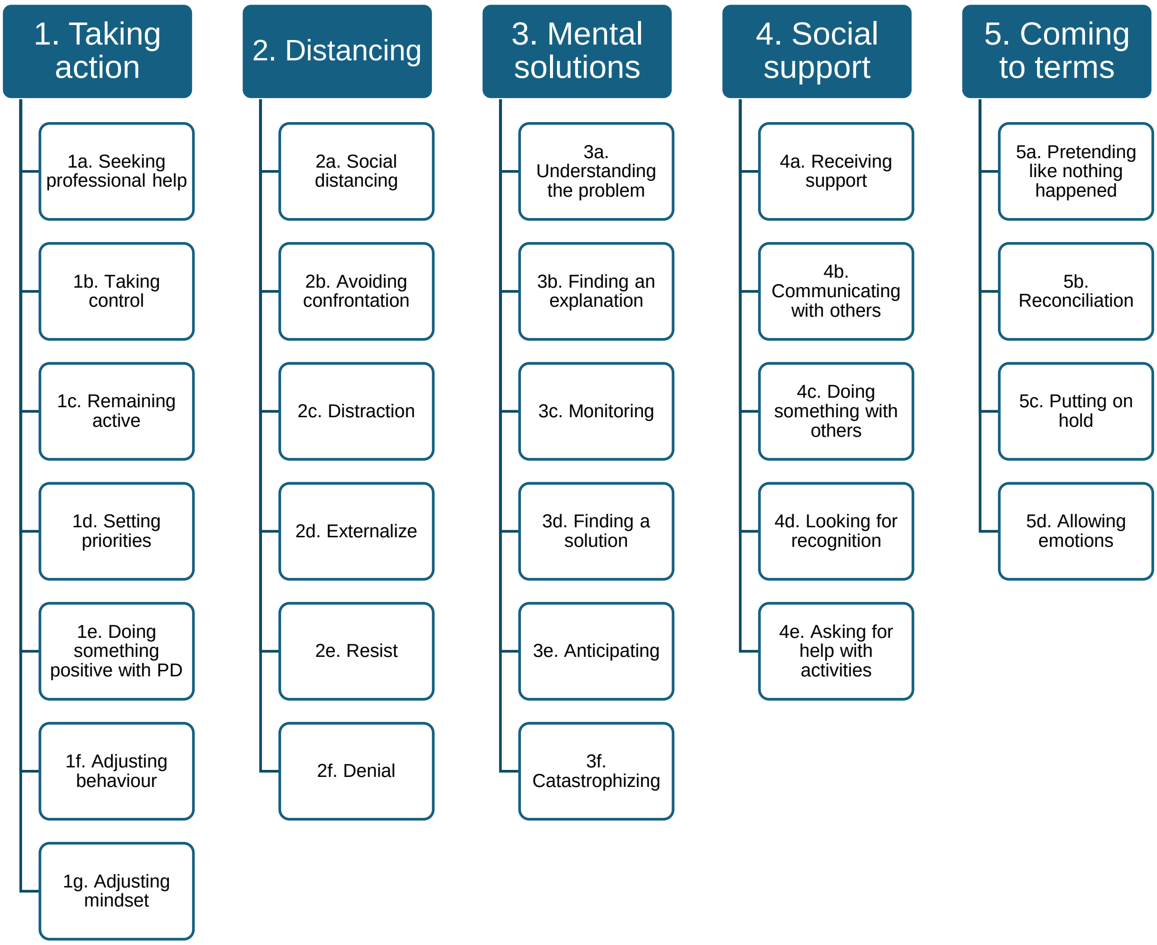

<u>1. Taking action</u>

Taking action is an active coping strategy, with efforts to positively change the situation. Often, these efforts are directed at the problem at hand, and therefore it is a predominantly problem-focused coping style.

All participants reached a point in time when they recognized that the health complaints that they experienced could not be solved by themselves. This included the moment that they noticed their first symptoms (in most cases motor), eventually leading to the diagnosis, but also looking for professional support in processing life events, **seeking help** from a physiotherapist to improve mobility, or going back to the neurologist to discuss advanced therapies. Furthermore, some participants took an active role in their treatment, **taking charge** in the process to receive care more suited to them or find a treating physician with a better personal connection, ask for a second opinion in cases of doubt, or actively discuss the timing for starting or increasing their medication regimen. Other participants stated their resistance to starting with medication due to their wish to stay independent, a fear to lose control when starting, uncertainty about the effect of the medication, or a currently acceptable quality of life and thus a self-perceived lack of benefits to start with medication. Moreover, participants took control in their working environment, setting clear boundaries with their employer and coming up with work-related solutions. Lastly, individuals took control in their social environment through

addressing situations that are undesirable for them, how their environment can better deal with these situations to suit the participants, and influencing the approach of interactions.
Another coping strategy was to **remain as active as possible**. Participants stated that they were afraid of losing the capability of doing activities once they quit them.
Some participants **changed their priorities** after receiving the diagnosis. Being aware of the progressive disease, they shifted their attention and efforts to the present. For some individuals, the life event of receiving an incurable disease made them reflect on their current priorities, putting more emphasis on their families instead of work or career ambitions. They decided to no longer prolong activities like long journeys but instead undertook these now while they were still in good health. For others, this new insight, or the loss of their job as a consequence of PD, prompted them to look for another purpose in life.
Another recurring theme was **to do something positive with their disease**. This included taking part in research, helping peers by sharing their experiences, or putting effort in fundraising for research for PD, putting a positive spin on a negative diagnosis.
Some participants actively **adjusted their behavior**. Participants started to take up an active lifestyle, putting lots of effort in becoming healthy and picking up a sport, as for some this was a way of taking back some control in the progression of the disease. Another way of changing their lifestyle included changing their diet to relief disease burden or medication interactions. A third major shift in behavior included applying the insights in changes in stimuli processing in their lives; they actively sought rest or a quiet place to regain energy, or to experience less stimuli. For some, this was a reason to reduce or stop working, to reduce stress and improve their health experience. In addition, structure in daily life was important for some participants, and although work might provide such a structure, others found regularity in exercise.
Lastly, **adjusting one's mindset** to improve their situations was a strategy to cope. One example of this is cognitive reframing; while recognizing that PD is a diagnosis with many implications, one participant called it a manual; after a period of not understanding the complaints that they had experienced, they finally understood where these complaints were coming from and how to deal with them. In such, the diagnosis was not as much of burden for them, but it provided guidelines on how to decrease the burden of their symptoms. Other participants reframed having PD as living a life with challenges instead of being a disease. Yet others tried to look for the silver lining, noting getting more close with their family as an positive outcome. Additionally, the use of humor was noted by participants as a way of coping to create a better atmosphere, causing them to speak more easily about their hardships, and removing social tension on this sensitive topic.
Moreover, being able to enjoy the "little things in life" was a common statement. Some participants stated living a more conscious life, being aware of the things and people around them. Others stated consciously reflecting on their identity, shifting for example from an emphasize on work to being a parent. Additionally, participants showed endeavors to increase their cognitive control: they mentioned focusing on the positives, but also being able to address their inner selves when they focus too much on negative thoughts or telling themselves that a period of pain will eventually end and that they have to endure. One participant noticed that negative thoughts worsened the (non)motor complaints, triggering him to actively shift the attention to the positive side of the situation. Lastly, participants found comfort and reassurance in the possibility for advanced treatments in a later stage.

2. Distancing

Distancing as a coping style includes mostly active ways of reducing the degree of having to deal with PD and can be both emotion- or problem-focused.

People with YOPD used **social distancing** as a coping strategy, by staying away from social events to avoid confrontation, shame or stimuli. Dyskinesias were a reason for social distancing because individuals both felt ashamed, but also believed their environment was ashamed of them. In addition, participants consciously avoided talking about PD to prevent others feeling sorry or misunderstanding them. Other reasons for social distancing included, e.g., that they do not want to be a burden to others, and avoiding shocking others with the diagnosis and hardships they endure. Lastly, social distancing included hiding symptoms, for example holding onto their arm to hide tremors.

Another distancing strategy was to **avoid confrontation** with their disease. Specifically for persons with YOPD, going to a peer event with a general, often older population with PD, frequently led to feelings of alienation and subsequent avoidance of such a confrontation of advanced disease. Additional rationales included being anxious of hearing symptoms they had not yet experienced and may - or may not - experience in the future. Some participants actively avoided activities that emphasized their limitations, causing them to, e.g., buy peeled vegetables, or to have family help in mobility instead of driving themselves. Besides that, participants frequently put up cognitive barriers, simply not wanting to think about PD every single day, avoid thinking about the future and its uncertainty, or to not give in to emotions such as feelings of sadness.

Another way of distancing was **externalization**; by placing hope in an external locus of control such as scientific advances, religion or fate, they did not have to actively interact with the disease.

Participants also sought **distraction** in activities like work, helping others or doing something creative.

Some participants showed **resistance** to the disease, experiencing the diagnosis as a fight, not wanting to accept the limitations of the disease.

Lastly, some participants actively **denied** limitations encountered, or even the disease itself. Although some state that they were not ready to deal with the consequences of the disease, this often led to participants overextending and going beyond their own limits. For instance, one participant stated having to regain energy by going to bed early in the weekend, forgoing fun activities with their family.

3. Mental solutions

Mental solutions are a (pro)active coping style about understanding the problem, coming up with solutions and anticipating on problems in the future. These strategies typically presented themselves in this sequential order.

Participants put effort into **understanding the problems** they encountered. An important aspect of this was to look for information on the internet, in books, but also by talking to peers. Specifically, some participants expressed their preference for information by peers, as this was more catered towards young persons and often more reliable than a search on the internet. They wanted this information to better understand the disease, medication, and prognosis. Looking for information remained important in all stages of the disease. Sometimes information seeking led to distress instead of providing reassurance. For instance, one participant in the diagnostic

phase found that brain tumors are an indication for brain imaging, which was insufficiently explained by their physician, causing anxiety. After the diagnosis, information was especially sought for major decisions such as starting with medication or starting with advanced therapies. Likewise, participants actively **sought explanations** of symptoms encountered during their own disease, in addition to looking for more general information. This was often described as a rational perspective on the problems encountered in daily life. For some, this resulted in self-blame, condemning themselves for diminishing relationships or regretting not mentioning side effects of medication to their healthcare providers.

In addition to this, participants wanted to keep a grip on their own symptoms by **monitoring** themselves. Participants applied this in daily life, reflecting on whether cognitive struggles are normal or part of the disease. Others valued outcomes of cognitive tests in research to compare their performance to the past, retaining some form of control on their cognitive function. Moreover, some participants were aware of obsessed behavior in the past, resulting in them actively monitoring their own behavior during activities. In contrast, others were wary of apathy.

Subsequently, many participants described **thinking of solutions** for the problems that they identified. For example, one participant actively sought peace of mind during a stressful situation to be able to better support their child.

More specifically, participants described a multitude of ways **to anticipate** on things to come, sometimes in response to prior experiences. These included arriving early to account for difficulties in mobility or urinary incontinence, or mentally preparing scenarios and coming up with possible reactions to them in advance. Participants often mention disclosing their diagnosis to prevent rumors and reduce stress during for instance a presentation. In contrast with distancing, some actively thought about the future, discussing this with their partner and children, but also being mindful of possible progression of disease with decrease in mobility, when buying a house. In some cases, participants considered euthanasia or even suicide if quality of life reached a very low point.

When thinking about and preparing for the future, some participants lost themselves in a negative train of thoughts, termed **catastrophizing**. One such spiral of negativity is demonstrated by a participant developing fear of falling in the early phase of disease. This caused her to avoid walking altogether, resulting in using a wheelchair in the first years of disease. After discussing this with a peer, she overcame her fear and started training to walk long distances, like pilgrimages. This confirmed that there was in fact no somatic reason for her to be limited to a wheelchair. Other participants were convinced that their disease would prove fatal soon, based on stories of much older patients. Some even started arranging their own funeral, believing their death to be imminent.

<u>4. Social support</u>

The social support included both passive and active ways of coping and was often emotion focused. Many participants stating feeling supported by their social circle, of which partners played a key role. However, friends, family, peers, colleagues, healthcare providers and teachers at a child’s school are all mentioned as sources of social support.

Receiving support was a passive way of achieving this effect. Participants received support through people being there for them, helping them to reflect on their limitations, sharing sadness, or taking away concerns that they might have. Some felt motivated by others to overcome their fears or to get active when they experience apathy or social anxiety.

Talking with others was another way to feel supported. Being able to share hardships such as physical limitations, non-motor symptoms, and discussing shared schedules added to a feeling of support. Interestingly, for some participants, sharing was one of the first things they did after being diagnosed, whereas others deliberately postponed this till they felt comfortable. Specifically in the case of discussing PD with children, some participants disclosed their diagnosis right away to allow them to ease into it, or to take away fears of them dying, whereas others deliberately did not tell their children until they were older to protect them from negative emotions or worries.

Third, participants stated that **doing an activity together with others** provided them with feelings of support. This included doing hobbies together with others, or shifting a focus of asking people to help in chores around the house to inviting them over for a social event and remaining socially involved.

For some participants, **seeking recognition** often provided them with feelings of support. Peers of similar age played an important role in this, especially given the unique and widespread challenges combined with the relative rarity of YOPD. Participants met peers during national events, in dedicated young onset Parkinson meetings in cafes, in support groups, or through (international) fora.

A more active coping style was implemented through **asking for aid**. This included asking for practical help in household chores, asking for aid in transport when mobility is decreased, or asking their families for help managing a day out with their children.

<u>5. Coming to terms</u>

Coming to terms is a passive style of coping, aimed at regulating themselves instead of solving the problem at hand, and therefore an emotion-focused coping style. It is often the path of least resistance to deal with a stressful situation.

One such strategy is ignoring the situation, or **pretending like nothing happened**. In order to continue living their life as usual, participants stated that they ignored selective symptoms or the diagnosis altogether.

Another strategy is **reconciliation**. Participants rarely used the word “acceptance” due to the progressive nature of PD; with the continuous threat of decline of function or manifesting of new symptoms, there was no perceived constant state that could be accepted. Some participants explained that they had accepted some aspects of disease, such as the fact that it is incurable, the uncertainty of the future, having to quit work, or diminishing of relationships. Furthermore, participants stated not being bothered anymore by bystanders staring, or not feeling shame anymore when they experienced dyskinesias. Participants recognized their lack of control in such situations.

A third way of coping was to **put some aspects of the disease on hold**. This was one strategy to reduce the time spent thinking or worrying about the disease.

Lastly, some participants acknowledged the disease but also the emotions that might come with it, **allowing space for these emotions** to be present instead of repressing them. Some showed a high degree of control in this, allowing themselves to be sad for a while, but to pick themselves up afterwards and carry on.
